# Supplementary material for: Effects of Cellulose Nanocrystals and Cellulose Nanofibers on the Structure and Properties of Polyhydroxybutyrate Nanocomposites
Source: Polymers (Basel). 2019 Dec 11;11(12):2063. doi: 10.3390/polym11122063 (PMC6960622; doi:10.3390/polym11122063)
Supplement: Supplementary file 1 [file polymers-11-02063-s001.pdf]

Supporting information

Supporting information1

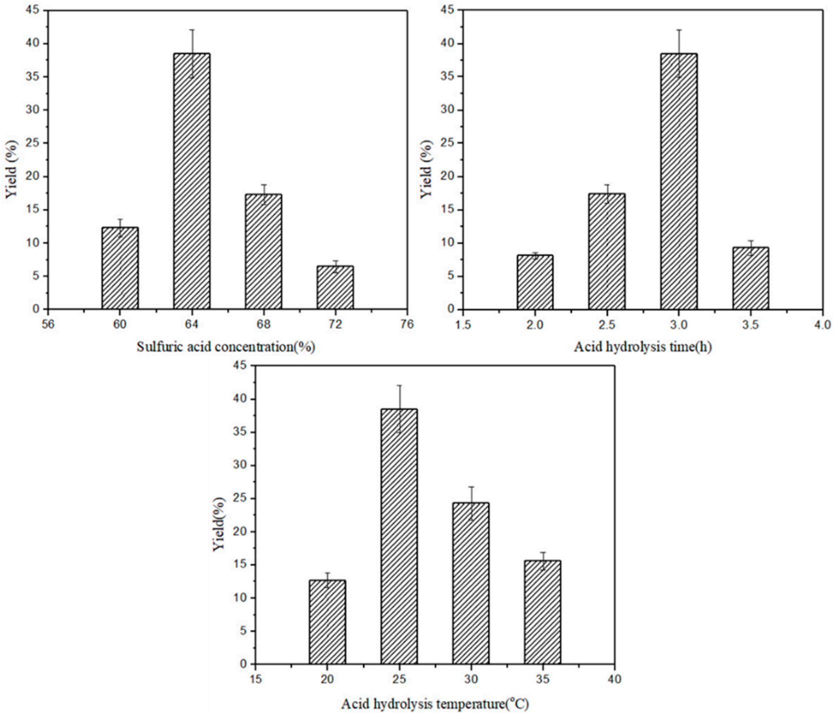

Figure S1. Effects of sulfuric acid concentration, acid hydrolysis time, and acid hydrolysis temperature on the yield of CNCs.

From the Figure S1, the best preparation process of nanocellulose can be obtained. The sulfuric acid mass concentration is 64%. The acid hydrolysis time is 3 hours, and the acid hydrolysis temperature is 25 °C. At this time, the yield of nanocellulose is 38.5%.

Supporting information 2

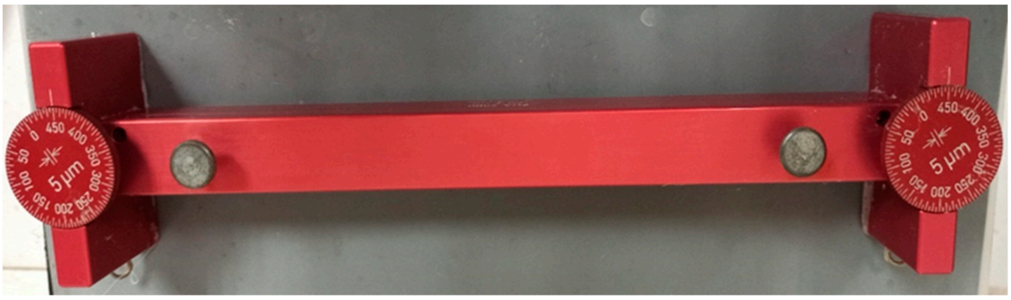

Figure S2. Machine stick on automatic film application machine.

As shown in the Figure S2, the automatic film application machine (ZEHNTNER, ZAA2300) was used to prepare the film, and thickness of the film by adjusting the stick height and the film speed on the film coating machine.

Supporting information 3

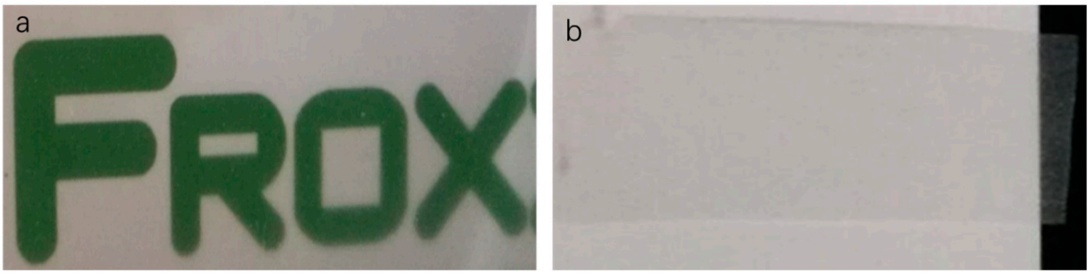

Figure S3. (a) PHB/CNCs film, (b) Actual picture of PHB / CNCs film fracture during mechanical test.

Some actual pictures of PHB/CNCs have been added and film fracture during mechanical testing to the supporting information. It can be seen from the Figure S3a that the surface of the composite film is flat, but bumps appear in some parts, which is caused by the aggregation of nanocellulose. Figure S3b shows that the composite film was uneven at the fracture during mechanical test fracture, which may be caused by the pull-out of the clusters of CNCs aggregated during fracture.
